# Regulatory Role of Fatty Acid Metabolism on Glucose-Induced Changes in Insulin and Glucagon Secretion by Pancreatic Islet Cells

**DOI:** 10.3390/ijms25116052

**Published:** 2024-05-31

**Authors:** Jorge Tamarit-Rodriguez

**Affiliations:** Biohemistry Department, Medical School, Complutense University, 28040 Madrid, Spain; tamarit@ucm.es

**Keywords:** pancreatic islets, insulin secretion, glucagon-secretion, starvation, glucose, fatty acids, norepinephrine, cGMP, cAMP

## Abstract

A detailed study of palmitate metabolism in pancreatic islets subject to different experimental conditions, like varying concentrations of glucose, as well as fed or starved conditions, has allowed us to explore the interaction between the two main plasma nutrients and its consequences on hormone secretion. Palmitate potentiates glucose-induced insulin secretion in a concentration-dependent manner, in a physiological range of both palmitate (0–2 mM) and glucose (6–20 mM) concentrations; at glucose concentrations lower than 6 mM, no metabolic interaction with palmitate was apparent. Starvation (48 h) increased islet palmitate oxidation two-fold, and the effect was resistant to its inhibition by glucose (6–20 mM). Consequently, labelled palmitate and glucose incorporation into complex lipids were strongly suppressed, as well as glucose-induced insulin secretion and its potentiation by palmitate. 2-bromostearate, a palmitate oxidation inhibitor, fully recovered the synthesis of complex lipids and insulin secretion. We concluded that palmitate potentiation of the insulin response to glucose is not attributable to its catabolic mitochondrial oxidation but to its anabolism to complex lipids: islet lipid biosynthesis is dependent on the uptake of plasma fatty acids and the supply of α-glycerol phosphate from glycolysis. Islet secretion of glucagon and somatostatin showed a similar dependence on palmitate anabolism as insulin. The possible mechanisms implicated in the metabolic coupling between glucose and palmitate were commented on. Moreover, possible mechanisms responsible for islet gluco- or lipotoxicity after a long-term stimulation of insulin secretion were also discussed. Our own data on the simultaneous stimulation of insulin, glucagon, and somatostatin by glucose, as well as their modification by 2-bromostearate in perifused rat islets, give support to the conclusion that increased FFA anabolism, rather than its mitochondrial oxidation, results in a potentiation of their stimulated release. Starvation, besides suppressing glucose stimulation of insulin secretion, also blocks the inhibitory effect of glucose on glucagon secretion: this suggests that glucagon inhibition might be an indirect or direct effect of insulin, but not of glucose. In summary, there seems to exist three mechanisms of glucagon secretion stimulation: 1. glucagon stimulation through the same secretion coupling mechanism as insulin, but in a different range of glucose concentrations (0 to 5 mM). 2. Direct or indirect inhibition by secreted insulin in response to glucose (5–20 mM). 3. Stimulation by increased FFA anabolism in glucose intolerance or diabetes in the context of hyperlipidemia, hyperglycemia, and hypo-insulinemia. These conclusions were discussed and compared with previous published data in the literature. Specially, we discussed the mechanism for inhibition of glucagon release by glucose, which was apparently contradictory with the secretion coupling mechanism of its stimulation.

## 1. Introduction

From the nutritional point of view, humans experience alternative phases of feeding and fasting conditions throughout the day. The carbohydrates and lipids ingested with a meal produce an increase in blood glucose and free fatty acids (FFAs) that impact pancreatic islet cells to change their hormone secretion. It is universally accepted that β-cells increase the secretion of insulin, whereas α-cells decrease that of glucagon. It is also generally agreed that these hormonal changes are mainly due to the elevation of blood glucose, whereas the FFA increase seems to have a less significant function. During fasting, glucose, FFAs, and insulin return to basal values (postabsorptive hours). When fasting is prolonged after the postabsorptive period, the body enters so-called early starvation [1]. Hepatic glycogenolysis is no longer enough to preserve a normal basal glucose, and FFAs are mobilized from adipose tissue: their metabolism in peripheral tissues (mainly muscle and kidney) inhibits glycolysis (FFA–glucose or Randle cycle, [2]) saving glucose to mainly feed the brain. After 24 h of no food intake, the organism enters an intermediate starvation period that lasts until about 24 days: it is characterized by increased liver and kidney gluconeogenesis, mainly from muscle amino acids [1]. In this period, the blood glucose concentration falls only by 30–35%, probably due to increased glucagon secretion, and then it remains relatively constant. After 24 days since its last meal, the human body enters the prolonged starvation period, and the metabolic rates of glucose (decreased utilization), FFAs (increased oxidation and ketone body production), and amino acids (used for increased gluconeogenesis) stabilize [1]. Insulin remains close to basal values, but β-cells lose their capacity to secrete insulin in response to glucose. This review will be focused on the potential mechanisms that FFAs might exert to modulate glucose-induced hormone secretion changes through their metabolism on both β- and α-cells.

## 2. Lipid Metabolism in β-Cells

### 2.1. Islet FFA Oxidation

Β-cells do not only significantly metabolize glucose, but also FFAs and L-α-amino acids. Islet FFA oxidation was studied in the pioneering work of C. Berne [3]. [U-^14^C]-palmitate oxidation to ^14^CO_2_ showed a hyperbolic relationship, with its concentration in the range from 0.25 to 2.0 mM. Human blood basal levels of FFAs (0.3 mM) already increase in the postabsorptive period (around 0.5 mM) and rise to values between 1 and 2.0 mM after 1 or 2 days of starvation [1]. The islet oxidation rate of 0.5 mM labeled palmitate in the presence of 3.3 mM glucose was 2.2-fold lower than that of 3 mM [U-^14^C]-glucose, and it was suppressed within 27% and 43% by 6 and 16.7 mM glucose, respectively [3,4]. The percentage suppression by 20 mM glucose was very similar (−62.2 ± 2.5) at three different palmitate concentrations (0.25, 0.50, and 1.0 mM). In addition, 0.25 mM 2-bromostearate (BrS), an inhibitor of carnitine palmitoyl transferase I (CPT I) catalyzing FFA transport trough the external mitochondrial membrane [5], caused a strong inhibition of 0.5 mM [U-^14^C]-palmitate oxidation to ^14^CO_2_ at different glucose concentrations (6.8-, 3.8-, and 2.8-fold at 3, 6, and 20 mM glucose, respectively) [3,4].

The rate of 20 mM [U-^14^C] glucose oxidation to ^14^CO_2_ was almost 30-fold higher than that of 0.5 mM palmitate oxidation at the same glucose concentration [4]. Taking into consideration the theoretical ATP yield obtained from the oxidation of 1 pmol of glucose (32 pmol) and 1 pmol palmitic acid (129 pmol), the calculated ATP production by glucose was around 7-fold higher than it was from palmitate.

Starvation for 48 h changed FFA metabolism of isolated rat islets considerably [4,6]. The rate of [U-^14^C]-palmitate oxidation to ^14^CO_2_ at 3 mM glucose was increased two-fold, compared with islets from non-starved animals. The 20 mM glucose failed to suppress the increased rate of palmitate oxidation in starved islets at fatty acid concentrations above the basal value [4,6]. It has been known for long time that starvation and diabetic state significantly diminish the content of malonyl-CoA (physiological inhibitor of carnitine palmitoyl transferase I (CPTI) derived from glucose) in the liver [7] and that the half-maximal concentration of the inhibitor on palmitate oxidation is higher in mitochondria from starved rats compared to fed rats [8]. Nowadays, it is known that AMP-dependent protein kinase (AMPK), a critical metabolic sensor, phosphorylates and inhibits the enzyme generating malonyl-CoA, acetyl-CoA carboxylase (ACC) [9,10]. Consequently, the balance between palmitate oxidation (catabolism) and esterification (anabolism) is strongly displaced in favor of oxidation. BrS (0.25 mM) did also strongly inhibit the upregulated and glucose-insensitive rate of palmitate oxidation in islets from starved animals at either 3 or 20 mM glucose concentrations [3,4].

The starvation-induced increase in islet palmitate oxidation did not affect either glucose utilization or oxidation that were not significantly modified by 0.25 mM BrS [4,11]. One might conclude that pancreatic β-cells, the major cellular component of pancreatic islets, are not under the control of the glucose–fatty acid or Randle cycle [2]. It seems that the β-cell is always ready to respond to an elevation in glucose concentration, independently of the nutritional state of the organism in question. This is beautifully shown by the works of Prof. D. McGarry’s group for both rats and humans: an elevated concentration of blood FFA is essential to recover the sensitivity of the β-cell to respond normally to glucose after a starvation period [12,13]. The higher the FFA and fatty-acyl-CoA concentrations, the higher the probability of overcoming the increased Ki of mitochondrial CPT I for malonyl-CoA in starvation. Perhaps changes in the lipid composition of the β-cell, induced by an unbalanced FFA catabolism/anabolism equilibrium, might be partially responsible for the suggested changes in CPT I enzyme activity.

**Figure 1:**
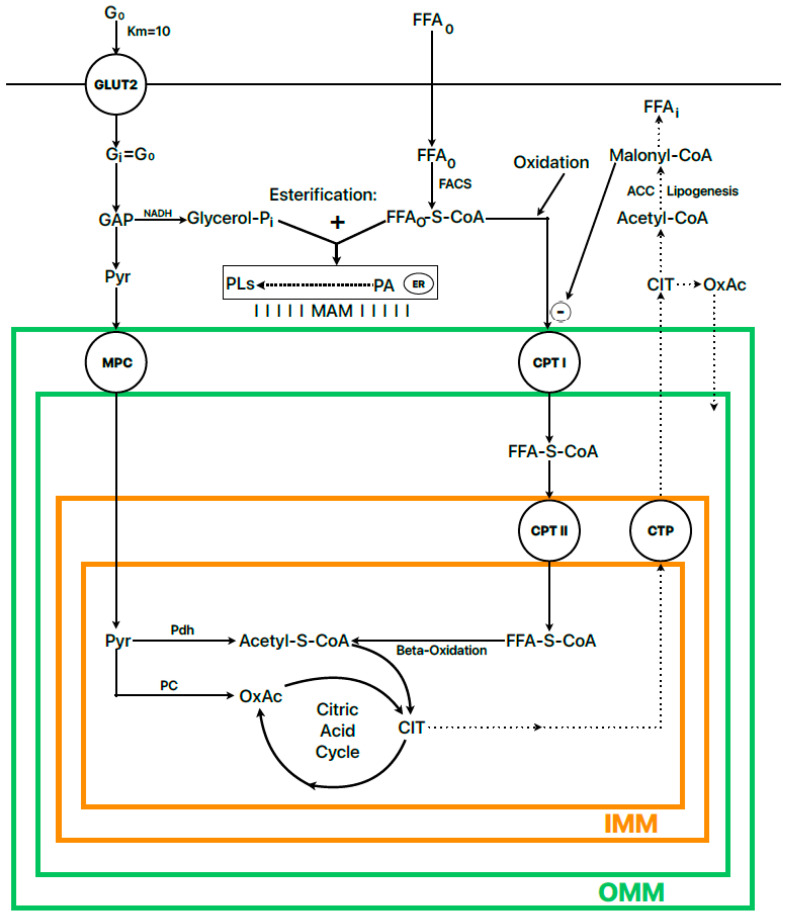
Scheme of the metabolic regulation of the balance between fatty acid esterification and oxidation regulated by the “fuel sensor” of acetyl-CoA carboxylase (ACC I and II). Citrate (CIT), phosphatidic acid (PA), phospholipids (PL^S^), fatty acids (FFAs), oxalacetate (OxAc), pyruvate (Pyr), glucose (G), carnitine palmitoyl transferase I (CPTI), fatty acyl-CoA synthetase (FAS), pyruvate dehydrogenase (Pdh), pyruvate carboxylase (Pc), mitochondrial pyruvate carrier (MPC), citrate transport protein (CTP), mitochondria-associated membranes (MAMs), inner mitochondrial membrane (IMM), outer mitochondrial membrane (OMM), endoplasmic reticulum (ER). The dotted line represents the lipogenesis pathway, which is weakly expressed in islet cells but is necessary to control the balance between FFA oxidation and esterification by malonyl-CoA. The broken line represents the branched pathway of phospholipid synthesis after the esterification of FFA to glycerol-3-phosphate. The subscripts “o” and “i” in G and FFAs refer to the extra- and intracellular concentrations of glucose and FFAs, respectively.

This view suggests further consequences: the two main substrates needed to maintain nutritional homeostasis, glucose and FFAs, must be reciprocally regulated, as their respective concentrations signal the predominance of either overall catabolism or anabolism, guided by glucagon and insulin, respectively. Given that β-cells are not subject to Randle cycle control, the amount of ATP generated by both substrates, glucose and FFAs, at their maximum concentrations is probably higher in the starved condition than the fed condition, and this apparently contradicts the established mechanism of the stimulus–secretion coupling of insulin secretion. This supports the view that FFAs, at variance with glucose, do not potentiate glucose-induced insulin secretion via their capacity to increase β-cell ATP production, but by modifying the lipid composition of the membranes of some intracellular compartments that facilitate signaling or distal exocytosis, as described below (Figure 1).

### 2.2. Islet Lipid Biosynthesis

According to the results of C. Berne [14], [U-^14^C]-glucose incorporation into triacylglycerols and phospholipids of lean ob/ob mouse islets was increased by 2.4- and 4.0-fold, respectively, at 17 mM compared to 3.5 mM glucose. The accumulation of labeled phospholipids was 2.6-fold higher than that of triacylglycerols. Data from our laboratory confirmed C. Berne’s general findings in rat islets [4,6]. They also showed that 20 mM glucose significantly stimulated the incorporation of 0.5 mM [U-^14^C]-palmitic acid into both triacylglycerols (+2.5-fold) and phospholipids (+2.0-fold) as compared with 3 mM glucose. Furthermore, 48 h of starvation suppressed 20 mM glucose-induced increase in labeled palmitic acid incorporation into TG and PL by 2.7- and 2.1-fold, respectively [4,15]. No effect of starvation was appreciated in islets incubated with 3 mM glucose. In addition, 0.25 mM BrS did not modify 0.5 mM labelled palmitate incorporation into TG or PL with 20 mM glucose in control fed islets, but it restored it to control values in starved islets [4].

The capacity of islets to perform lipogenesis was studied, recording the incorporation into several lipid fractions (PL, TG, diacylglycerol, or DG, as well as FFA) of 3 and 20 mM [U-^14^C]-glucose [6] in the presence of unlabeled palmitate. PL labelling increased linearly with the glucose concentration from 3 to 20 mM glucose, and the glucose response was potentiated by a range of unlabeled palmitate concentrations (1.9-, 4.0-, 4.3-, and 8.4-fold at 0.0, 0.25, 0.5 and 1.0 mM palmitate, respectively).

Labelling of the other lipid fractions reached a steady level before the two hours of incubation. TG labelling in response to a change in glucose concentration from 3 to 20 mM showed a similar dose–response curve to potentiation by palmitate. However, it was much lower than PL labelling: the minimum and maximum incorporation were 4.2-fold lower. The labelling of islet DG and FFA fractions were still weaker than that of TG. There was no palmitate potentiation of either DG or FFA fractions at 3 mM glucose, but DG label incorporation was significantly stimulated by 20 mM glucose (around 2-fold). These results suggest that lipogenesis is not significantly contributing to the biosynthesis of islet lipids; glucose seems to contribute mainly by supplying D-glycerol phosphate for the esterification of incoming FFAs, as was already suggested by C. Berne years ago [14]. A low expression of the lipogenesis pathway in β-cells might be compatible with a regulatory role of acetyl-Coa synthetase on the mitochondrial transport of incoming extracellular FFAs. Another relevant conclusion is that a strong correlation could be demonstrated between islet secretory rates and labelling of islet PL by [U-^14^C]-glucose in the absence and presence of palmitate and in islets from either fed or starved rats [6].

Continuing on, 48 h of starvation induced a severe change in [U-^14^C]-glucose incorporation into islets’ lipids. The 3 mM [U-^14^C]-glucose labelling of islet PL was reduced by 41.1 ± 6.0% at all palmitate concentrations tested [15]. With 20 mM glucose, PL labelling decreased inversely to the unlabeled palmitate concentration (−44.0, −20.0, −23.0, and −15.0%, at concentrations of 0.0, 0.25, 0.5, and 1.0 mM palmitate, respectively). Are the higher palmitate concentrations counteracting the higher Ki of malonyl-CoA for inhibition of CPT I during starvation, as already mentioned above? TG labelling showed no change from starvation at 3 mM glucose, nor was it decreased at any unlabeled palmitate concentration except for the highest one, 1.0 mM [6]. Meanwhile, islet FFA labelling was significantly decreased under all conditions tested. DG lipid fraction, like TG, was only suppressed by starvation at concentrations of palmitate higher than 0.25 mM.

### 2.3. Islet ^45^Ca^2+^-Turnover and Phospholipid Biosynthesis

Motivated by the tight correlation between insulin secretory rates and labelling of the bulk of islet PL by [U-^14^C]-glucose [6], a correlative study on the labelling of various PL fractions with islet ^14^Ca^2+^-uptake was performed [15]. A time-kinetic study showed that 20 mM [U-^14^C]-glucose increased the labelling of all PL fractions several-fold, as compared to 3 mM labelled glucose. Isotopic equilibrium was reached in phosphatidate (PA), polyphosphoinositides (PPIs), and diacylglycerol (DG) between 5 and 15 min, as well as triacylglycerol (TG), phosphatidylethanolamine (PE), and phosphatidylinositol (PI) between 60 and 120 min. In addition, phosphatidylcholine (PC) showed a constant rate of labelling. Furthermore, 20 mM [U-^14^C]-glucose incorporation into every phospholipid fraction was potentiated approximately 2-fold by increasing palmitate concentration from 0.25 to 1.0 mM palmitate. Omission of extracellular calcium or trifluoperazine (calcium–calmodulin inhibitor) reduced labelled glucose incorporation into DG, TG, and neutral but not acidic phospholipids [16]. The calcium sensitivity of de novo synthesis of glycerophospholipids might be due to the calcium dependence of their own precursor enzyme, phosphatidate phosphohydrolase [17,18]. An increased rate of PPI synthesis is compatible with their role in the release of calcium from endoplasmic reticulum stores: 20 mM glucose significantly increased the islet production of IP_3_ with respect to 3 mM, although it was not affected by the presence of 0.25 mM palmitate [19]. Islets from rats that were starved for 48 h incorporated significantly less labelled glucose at 1.0 mM palmitate in all the lipid fractions [15].

Does palmitate potentiation of labelled glucose incorporation into phospholipids have any effect on ^45^Ca^2+^-turnover into a lanthanum-nondisplaceable pool [20] of rat islets? The uptake of ^45^Ca^2+^into this pool was linearly stimulated more than 4-fold during the initial 5–15 min. After 120 min, the increase in ^45^Ca^2+^-uptake at a 20 mM glucose concentration was 11.4-fold higher than at 3 mM [15]. Addition of palmitate (either 0.25 or 1.0 mM) potentiated the initial (15 min) rate of uptake induced by a 20 mM concentration 2.1-fold, but not the ^45^Ca^2+^-content after 120 min incubation, suggesting that palmitate accelerates ^45^Ca^2+^-turnover of the same pool as glucose. Palmitate (1 mM) also significantly increased the efflux rate of ^45^Ca^2+^ from islets preloaded with the isotope for 120 min at 20 mM glucose and then washed in nonradioactive medium. Islets from rats starved for 48 h showed a reduced short- (15 min, −2.1-fold) and long-term (120 min, −2.5-fold) ^45^Ca^2+^-uptake at 20 mM glucose in the absence and presence of 1 mM palmitate. This reduction was completely reversed by two fatty acid oxidation inhibitors, BrS (0.25 mM) and 2-tetradecylglycidate (10 µM), without affecting the ^45^Ca^2+^-uptake of control (fed) islets. FFA incorporation into some islet phospholipids may facilitate the uptake of Ca^2+^ into β-cells or its release from the endoplasmic reticulum [21].

### 2.4. Stimulation of Islet Protein Kinase C Translocation through Palmitate Metabolism

The potentiation of DG biosynthesis at high glucose might also contribute to the potentiation of the insulin secretory response by activating protein kinase C activity [19]. At a 3 mM glucose concentration, 64% of the total enzyme activity was in the soluble fraction, as measured by the incorporation of [^32^P] orthophosphate from [γ^32^P] ATP into a synthetic peptide from the myelin basic protein (MBP 4–14) [19]. A concentration of 20 mM glucose alone did not modify this basal distribution of PKC activity, but the addition of 0.25 mM palmitate increased the percentage of the particulate fraction from 41 to 49% (*p* < 0.001). The 2 mM hydroxy citrate (ATP citrate lyase inhibitor) significantly suppressed the particulate fraction activity, and phorbol-12,13-dibutyrate (20 ng/mL) exerted the most potent translocation to the particulate fraction (71%, *p* < 0.001).

### 2.5. Modulation of Glucose-Induced Insulin Secretion via FFA Metabolism

The insulin secretory response of batch-incubated rat islets to 20 mM glucose, as compared to 3 mM glucose, was significantly potentiated in the same range of palmitate concentrations used in metabolic experiments (7-, 10-, 6-, and 9-fold, at 0.0, 0.25, 0.5, and 1.0 mM palmitate) [6]. After 48 h of starvation, the insulin secretory response to 20 mM glucose plus palmitate was consistently suppressed (−3.6, −3.6, −3.8-, and −6.6-fold at 0.0, 0.25, 0.5, and 1.0 palmitate). The insulin response of control fed islets to 20 mM glucose in the absence of palmitate was suppressed 2.8-fold after 48 h of starvation and fully recovered by 0.25 mM BrS [4]; BrS did not alter the stimulation of insulin secretion in control fed islets. 1 µM trifluoperazine induced a similar reduction in 20 mM glucose stimulation of insulin secretion as calcium omission from the incubation medium (−70%), without affecting ^45^Ca^2+^-uptake [16]. The percentage suppression of calcium omission on glucose (20mM)-stimulated secretion was not significantly altered by the presence of 0.25 or 1.0 mM palmitate [16].

### 2.6. Norepinephrine Inhibition of Insulin Secretion

Norepinephrine is known to be an important regulator of whole-body metabolism, and it exerts a potent inhibitory action on glucose-induced insulin secretion through the specific activation of α_2_-adrenergic receptors in pancreatic islet β-cells [22]. It has been shown to have multiple targets in β-cells, like activation of K_ATP_-channels, inhibition of adenylyl cyclase, and a more “distal” effect on the stimulus–secretion coupling mechanism of glucose-induced insulin secretion [23]. Some studies have also reported a suppression of L-type Ca^2+^channels in tumoral transformed β-cells [24,25]. Considering the known capacity of norepinephrine to stimulate lipolysis in adipose tissue and to inhibit enzymes implicated in the biosynthesis of phospholipids [26,27], we have investigated whether the amine could reproduce some of the effects of starvation on islet palmitate metabolism and ^45^Ca^2+^-uptake [28].

Glucose (3 to 20 mM) stimulation of insulin secretion was dose dependently inhibited by norepinephrine (10^−9^ to 10^−6^ M) with an IC_50_ of 10^−7^ mM. Yohimbine (10^−5^ mM) alone, an α_2_-antagonist, slightly stimulated insulin secretion with 20 mM glucose and completely recovered the insulin secretion from 10^−6^ mM norepinephrine inhibition. The drug, within the whole range of concentrations used, did not exert any modification on basal insulin release with 3 mM glucose.

The initial uptake of ^45^Ca^2+^ in a lanthanum-nondisplaceable pool of rat islets was linearly stimulated more than 5-fold by 20 mM glucose, as compared with 3 mM, and reached an apparent steady level after 15 min [28], always in the presence of 1 mM palmitate. Norepinephrine (10^−6^ M) decreased glucose-stimulated ^45^Ca^2+^-uptake to levels overlapping those of 3 mM glucose. Half the maximum inhibition of short- and long-term inhibition of ^45^Ca^2+^-uptake was reached at 10^−7^ M norepinephrine. Yohimbine (10^−5^ M) did not exert any effect by itself, but it almost completely blocked norepinephrine-induced inhibition of either short- and long-term ^45^Ca^2+^-uptake stimulation by glucose. In the absence of palmitate, the percentage increase in the initial ^45^Ca^2+^-uptake by 20 mM glucose with respect to 3 mM was two-fold lower than in the presence of palmitate, but the steady content was almost identical, and it was similarly suppressed by 10^−6^ M norepinephrine.

Norepinephrine (at 10^−7^ and 10^−6^ M) dose dependently suppressed 20 mM [U-^14^C]-glucose incorporation into either neutral glycerolipids or acidic phospholipids. Its suppressive effect at 10^−6^ M was completely blocked by 10^−5^ M yohimbine.

It has already been commented above that labelled glucose incorporation into all islet lipidic fractions is very significantly suppressed by 48 h of starvation in islets from starved rats. Therefore, it was interesting to check whether norepinephrine, like starvation, suppressed islet lipid biosynthesis by favoring fatty acid oxidation over esterification [28]. The experimental data clearly show that norepinephrine (at 10^−7^ and 10^−6^ M) induced a highly significant decrease in [U-^14^C]-palmitate oxidation to ^14^CO_2_ in both fed islets and islets starved for 48 h with 3 mM glucose, which was almost completely blocked by 10^−5^ M yohimbine. With 20 mM glucose, the rate of palmitate oxidation of starved islets was almost 6-fold higher compared to fed islets; norepinephrine (at 10^−7^ and 10^−6^ M) suppressed it almost completely, and the amine effect was blocked by yohimbine. It may be concluded that, in this case, a potentiation of islet palmitate oxidation is not the cause, like in starvation, of norepinephrine-induced suppression of islet de novo lipid synthesis. The more plausible explanation for this difference in islets is an inhibition of fatty acyl-CoA synthetase (FAS), like what has been previously confirmed in adipocytes [27]. FAS is responsible for FFA carboxyl group activation through its transfer to HS-CoA, which primes FFAs to either be transported to mitochondria by CPT1 for oxidation or to be esterified to glycerol phosphate for complex lipid biosynthesis.

### 2.7. α2-Adrenergic Signaling Responsible for Norepinephrine Effects on Islet FFA Metabolism and Their Consequences for Insulin Secretion

There seems to be a consensus that all known effects of norepinephrine contributing to its inhibition of insulin secretion are mediated by the activation of pertussis toxin-sensitive G_i_ and G_o_ proteins, which then increase or decrease the intracellular synthesis of a second messenger via a specific effector [22]. It is also known that a single occupied receptor can interact with more than one G-protein. One postulated mechanism to explain the mechanism of norepinephrine inhibition of insulin secretion is suppressing glucose stimulation of cAMP through inactivation of β-cells’ adenylyl cyclases [22]. As the addition of dibutyryl cAMP [29] or increasing its intracellular concentration with forskolin [30] does not reverse norepinephrine inhibition, the comparative effects of the two second messengers, cAMP and cGMP, were tested on three parameters when stimulated by glucose alone and when stimulated together with palmitate [31]:

1. The effects of both messengers were assayed in incubated rat islets in a range of five concentrations (0 to 1 mM) on the insulin secretory response to 20 mM glucose at three palmitate concentrations (0, 0.25, and 1.0 mM). cAMP significantly increased the insulin response to glucose at 0.25, 0.50, and 1.0 mM in the absence and presence of 0.25 mM palmitate, but not of 1 mM. cGMP significantly inhibited insulin secretion from 0.25 onwards at the three palmitate concentrations. The simultaneous addition of 0.25 mM cGMP and 1 mM cAMP did not reverse cGMP-induced inhibition with either 0 or 1.0 mM palmitate [31].
2. D-[U-^14^C] glucose incorporation into islet lipids (DG, TG, PA, PI, PPI) in the absence of palmitate was not modified by the presence of 1 mM 8-Br-cGMP with either 3 or 20 mM labeled glucose. In the presence of 1 mM palmitate, 1 mM 8-Br-cGMP significantly suppressed 20 mM labeled glucose incorporation into all the lipid fractions analyzed; the decrease in labeled incorporation was not recovered by the simultaneous addition of 1 mM 8-Br-cAMP [31].
3. No effect was appreciated by either of the two messengers on the initial (15 min) ^45^Ca^2+_^uptake for 3 mM glucose. In addition, 0.25 mM 8-Br-cGMP did not modify the initial uptake of calcium stimulated by 20 mM glucose alone. The addition of 1 mM palmitate to 20 mM glucose potentiated around 2-fold glucose stimulation, and in the presence of 0.25 mM cGMP, the potentiation capacity of palmitate was almost completely suppressed. 1 mM 8-Br-cAMP was unable to recover 0.25 mM 8-Br-cGMP inhibition of palmitate potentiation at 20 mM glucose [31].

### 2.8. Norepinephrine Stimulation of Islet Adenylyl and Guanylyl Cyclase’s Activities [31]

A dose–response range of norepinephrine concentrations was used (0.01, 0.1, and 1.0 µM). At 3mM glucose, norepinephrine significantly stimulated islet plus medium cGMP content at all amine concentrations independently of the presence of 1 mM palmitate. An almost identical response was observed with 20 mM glucose. Independently of the glucose concentration (3 or 20 mM), 10^−5^ M yohimbine did not modify basal (absence of norepinephrine) islet cGMP content but almost completely suppressed its stimulation via 10^–6^ M norepinephrine at 5 and 60 min. Pertussis toxin is an enzymatic activity responsible for the ADP-ribosylation of G proteins from NADP^+^ causing inactivation of G-protein function. At 3 µg/mL, it almost completely blocked the elevation of islets + medium content triggered by 1 µM norepinephrine after 5 and 60 min of incubation. It also blocked the small increase in cAMP triggered by 20 mM glucose. Nitroprusside (activator of soluble guanylyl cyclase) [32] significantly stimulated islet production of cGMP at both the 10 and 100 µM concentrations, but it had no effect on cAMP [31].

There are three important differences between the behavior of cAMP and cGMP in islets stimulated by norepinephrine. One is the time-kinetics after stimulation with 10^−6^ M norepinephrine: whereas islet cGMP content accumulated between 5 and 60 min of incubation, generating a 4-fold increased content in the nucleotide, cAMP content decreased 1.7-fold in the same time scale. Secondly, the basal content of cGMP was around 32-fold higher than cAMP. Thirdly, cGMP content was already significantly increased after 5 min of stimulation with 10^−6^ M norepinephrine, whereas cAMP was significantly decreased. This difference was strongly amplified at 60 min (+190-fold in favor of cGMP). One characteristic in common with both cyclic nucleotides was that their respective stimulation (cGMP) and suppression (cAMP) was canceled by pertussis toxin. These experimental results demonstrate that islet palmitate metabolism is also under control of α2-adrenergic receptors whose agonists exert an inhibitory effect through the activation of a yet-unknown G-protein mediating the stimulation of a guanylyl cyclase. More recent reports have supplied proof that the inhibition of both Ca^2+^-uptake stimulated by a VDCC agonist (BAY K 8644) and the cAMP response to forskolin (an adenylyl cyclase stimulator) were blocked by previous incubation with pertussis toxin in HIT cells and rat islets [24,33]. In parallel experiments, the authors showed that the stimulation of insulin secretion by BAY K 8644 and glucose was also similarly blocked, and their study of specific ADP-ribosylation of G-proteins pointed to the implication of a G0α subunit. A recent study of the functional consequences of specific knockouts of a series of Gα -subunits in mice demonstrated that the G02 (G02α subunit) protein is a unique transducer of the agonist stimulation of α2-adrenergic receptors [34]. Russel M.A. and Morgan N.G. have identified the presence of the soluble and three particulate isoforms of the known guanylate cyclase’s (pGC-A, pGC-B, and pGC-C) in BRIND-BD11 β-cells [35]. PGC-C is activated by guanylin peptides produced by intestinal epithelial cells also present in the pancreas [36]: guanylin and uroguanylin blunted palmitate-induced insulin secretion, and the increase in cellular TG in RIN 5mF β-cells chronically stimulated with palmitate prevented β-cell steatosis, besides other effects [37]. One wonders whether norepinephrine is also activating PG-C in the pancreatic islet β-cell population.

### 2.9. ER–Mitochondrial Interactions (ER–Mito)

**Figure 2:**
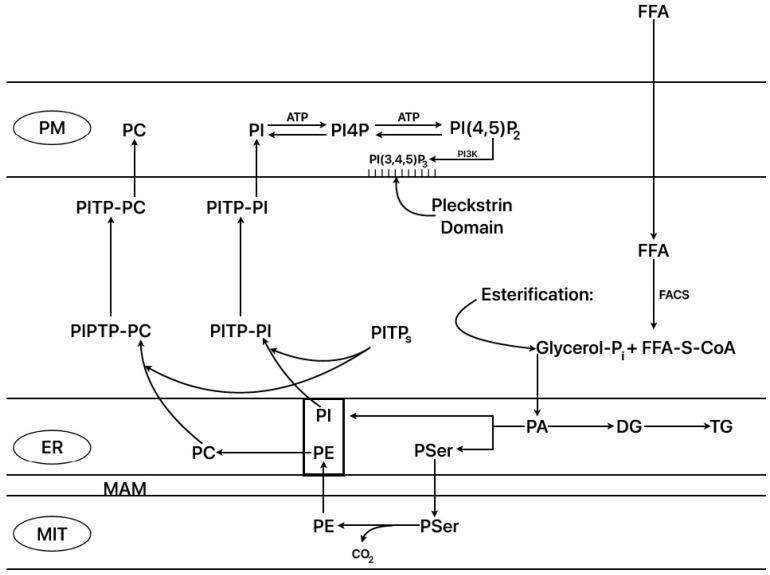
Transfer of phospholipids from the endoplasmic reticulum (ER) to the plasma membrane. Phosphatidylinositol transfer proteins (PITPS), phosphatidic acid (PA), phosphatdylinositol (PI), phosphatidylcholine (PC), phosphatidylserine (Pser), phosphatydil ethanolamine (PE), diacylglycerol (DG), triglycerides (TGs), tatty acyl-CoA synthetase (FAS), mitochondria-associated membranes (MAMs), mitochondria (MIT), endoplasmic reticulum (ER), plasma membrane (PM). Pleckstrin domain is a section of the inner leaflet of the plasma membrane rich in phosphatydilinositol(3,4,5)trisphosphate (PI(3,4,5)P3), to which different effectors of membrane-activated receptors (insulin receptor) bind to further generate intracellular messengers.

It is known that synthesis of the more abundant phospholipids of the inner mitochondrial membrane (PE and PC) takes place in specific locations in the endoplasmic reticulum associated with the mitochondria (mitochondria-associated membranes, or MAMs) and then is transferred to the mitochondria [38]. A positive correlation between mitochondrial ATP and PE contents in hepatoma cells has been demonstrated, together with an increased respiratory rate after downregulation of phosphatidylethanolamine N-methyltransferase (PEMT) that results in the elevation of mitochondrial PE content [39]. Fasting triggers the so-called “adaptive response to fasting” [40] that moderates FFA oxidation, stimulating the synthesis of the enzyme methionine S-adenosyl transferase (MAT I) in the mitochondria-associated membranes (MAMs) of the endoplasmic reticulum tethered to the mitochondria (ER–mitochondria junctions). MAT I catalyzes the trimethylation of PE to PC at the expense of S-adenosyl methionine in the major organ systems, liver, brain, and adipose tissue. Small variations in the PC/PE molar ratio may contribute to the development of non-alcoholic fatty liver disease (NAFLD) in humans, and ratios below 1 may induce liver failure [41]. To our knowledge, its activity and/or function have not yet been explored in pancreatic islets. Anyhow, it has been shown that starvation of rats for 48 h significantly suppressed the islet amount of PC and PE by 3- and 2-fold, respectively, suppressing their corresponding ratio by 54% in comparison with control fed islets [15]. Moreover, it is known that the main phospholipids (PC, PI) synthesized in the ER are transferred to the plasma membrane by means of the “phosphatidyl inositol transfer proteins” (PIPT α, β, and more) (Figure 2) [42]. Plasma membrane PI is repeatedly phosphorylated by PI4-kinase and PI4P-5kinase to PI(4,5)P_2_. Phospholipase C activation occurs via G-protein-coupled receptors or via increases in cytosolic Ca^2+^ in the micromolar range, hydrolyzing PI(4,5)P_2_ to I(1,4,5)P3 (IP3) and DG. These two messengers and their precursors are rapidly depleted and have to be replaced by importing PI from the ER via some PITP forms. Readers interested in more detailed information about the cellular cyclic system that regulates the replenishment of PI and its two main phosphorylated species at the plasma membrane may consult reference [42]. There is also information on the possible roles of PI phosphorylated forms on exocytosis and ion channel regulation [43]. PITP, PI(4,5)P_3_, and other lipids (FFA, PA, cholesterol) have been implicated in post trans-Gogi network secretory vesicle formation (vesicle budding and fission) and their docking and priming at the plasma membrane [44,45].

Besides its participation in the “adaptive response to starvation” in the maintenance of membrane lipid composition [40], ER–Mito coupling also contributes to regulating cellular calcium homeostasis and mitochondrial bioenergetics, two tightly connected processes [38]. The mitochondrial part of ER–Mito contacts possesses a “mitochondrial calcium uniporter” (MCU) of low affinity for Ca^2+^ (Kd = 15–20 µM), energized by the negative electrochemical gradient through the inner mitochondrial membrane (V_i_–V_e_~−180 mV). The cytosolic calcium ranges from 50 to 100 nM at rest and peaks between 1 and 3 µM under stimulation. The unfavorable kinetics of MCU seems to be overcome by the very close apposition of MCU to a “constitutive” inositol 1-4-5-triphosphate (IP3) receptor type 3 (IP3R-3) of ER [46], generating high [Ca^2+^] concentration cytosolic microdomains that allow a fast mitochondrial calcium uptake [38]. Under cell activation, mitochondrial Ca^2+^ rises, generated by IP3-dependent ER Ca^2+^ release, indirectly stimulating key Krebs cycle regulatory enzymes (isocitrate dehydrogenase, oxoglutarate dehydrogenase), NADH import from cytoplasm (FAD-dependent glycerol phosphate dehydrogenase), and some mitochondrial membrane carriers, as well as directly stimulating the electron transport chain and F0F1 ATP synthase activity [38]. This results in the stimulation of mitochondrial respiration and the production of ATP.

Cells respond to a decrease in cellular ATP through activation of catabolic pathways and inhibition of anabolic ones, or via activation of autophagy [47]. Activation of AMP-activated protein kinase (AMPK) plays a fundamental role; it inhibits the ACC 1 enzyme responsible for the synthesis of malonyl-CoA, which restricts mitochondrial FFA oxidation [9,10]. This results in the predominance of FFA oxidation over their esterification, supplying a substrate to feed mitochondrial respiration. A steady basal Ca^2+^ transfer from ER to mitochondria seems to be essential for the maintenance of bioenergetically active mitochondrial function [47]. In more extreme conditions, like the failure of mitochondrial signaling by IPR-3, it triggers autophagy that recycles cytoplasmic constituents to keep mitochondrial respiration on. AMPK activation is reinforced by phosphorylation as well as the phosphorylation and inhibition of its substrate ACC 1 [47]. Although the precise role of AMPK phosphorylation seems to not be fully understood, autophagy is not dependent on mTOR phosphorylation [47].

Finally, an excessive matrix calcium accumulation (Ca^2+^ overload) resulting from a long-term cellular stimulation leads to a “permeability transition” of the inner mitochondrial membrane mediated by a Ca^2+^-sensitive, high-conductance, and voltage-dependent channel, the membrane transition pore (mPTP). This channel allows the diffusion of small molecules (up to 1500 Da) and provokes mitochondrial depolarization, impairs oxidative phosphorylation, and increases ROS production [38]. In the end, mitochondrial swelling and outer mitochondrial rupture facilitates release of pro-apoptotic factors and the assembly and activation of the “apoptosome” (see Ref. [38] for deeper details).

A model has been proposed for the “time-dependent regulation of ER-Mito communication by glucose” in β-cells: in the face of an acute positive stimulation of MAM function, a chronic one empties the ER Ca^2+^ that may generate ER stress and mitochondrial fission [48]. Chronic stimulation with the high glucose of INS-1 cells and human pancreatic islets (48 and 72 h) reduces acute glucose stimulation of insulin release and insulin content, accompanied by a decrease in ER Ca^2+^stores and an increase in basal mitochondrial Ca^2+^content, probably due to a block of ER–Mito calcium transfer [48]. It is accompanied by signs of ER stress and mitochondrial dysfunction (mitochondrial fragmentation).

A significant decrease in the number of IP3R2-VADC-1 complexes was detected in β-cells of diabetic islets via in situ proximity ligation assay [49]. The authors also found a significant reduction in ER-Mito contacts in MIN6-B1 cells incubated with 200 µM palmitate and significantly increased pro-apoptotic pathways. However, the palmitate effects should be considered with some caution due to the high palmitate/BSA = 6 ratio used in the experiments.

### 2.10. Summary and Physio-Pathological Implications

Previous experimental trials aimed at confirming that palmitate oxidation contributes to the stimulation of insulin secretion were not successful [50,51,52]. Caution should be taken to restrict the BSA/FFA ratio to a value close to that found in plasma: around 3 at 2 mM palmitate. It is also convenient to remind readers that etomoxir (an irreversible inhibitor of mitochondrial CPT I enzyme) has some off targets at higher than 5 µM, affecting oxidative metabolism of substrates different from FFA and interfering with respiratory complex I [53,54]. The suppressive effect of fasting/starvation on glucose-stimulated insulin secretion has been mainly explained as a failure of some steps of islet glycolysis or as diminished glucose signaling through adenylyl cyclase. However, no cause-and-effect relationship has yet been found between decrease in glucose metabolism and starvation: 1. ATP content of fasted islets was increased despite a decreased rate of glucose utilization [55]. 2. Glucokinase activity was suppressed (within 18–30%), as well as phosphofructokinase, although the starvation effect on the insulin response to glucose was not compared [56]; glucose use was decreased within 23% and glucokinase activity in islet homogenates within 31%, whereas the insulin secretory response after 3 days of starvation was completely suppressed [57]. Glucose or palmitate oxidation were not studied in these mentioned reports. 3. The other main reason argued for explaining the suppression effect of fasting/starvation on insulin secretion is a diminished islet cAMP response to a stimulatory glucose concentration [58,59,60,61]. As adenylyl cyclase is stimulated at suprathreshold glucose concentrations for insulin secretion, its failure under fasting/starvation should be correlated with a suppression of glucose metabolism, unless glucose itself is directly stimulating cAMP production. By contrast, our main conclusion is that an exacerbated and uncontrolled mitochondrial FFA oxidation was the main cause of the suppression of glucose-stimulated secretion via fasting/starvation, for the reasons discussed below.

Fasting/starvation provides a physiological condition that remarks on the importance of FFAs in the co-regulation of insulin secretion by β-cells together with glucose. FFAs do not behave like the traditional metabolic stimulus of insulin secretion, which is expected to be catabolized trough mitochondrial oxidation to generate ATP. They can reinforce the insulinotropic action of glucose through their anabolism to the synthesis of complex lipids. Given that β-cells are not subject to Randle cycle control, the amount of ATP generated by both substrates, glucose and FFA, at stimulating and potentiating concentrations, respectively, during fasting/starvation, is probably above control fed conditions, which apparently contradicts the established mechanism of the stimulus–secretion coupling of insulin secretion by metabolic stimulators. This was confirmed by the complete recovery of the insulin response to glucose of isolated islets from starved rats via palmitate oxidation inhibitors (2Br-S, tetradecylglycidate, etomoxir). This in vitro evidence is supported by human studies showing that plasma FFAs are essential for recovering the insulin response to glucose [13]. Moreover, palmitate does not potentiate glucose-induced insulin secretion at basal concentrations but requires a slightly higher (6 mM) [4] level to allow the generation of enough malonyl-CoA to partially inhibit fatty acid oxidation.

Poor β-cell capacity for lipogenesis makes them very dependent on plasma FFA concentrations. Therefore, during fasting/starvation, the inhibition of acetyl-CoA carboxylase (ACC, generator of malonyl-CoA, see above) determines the predominance of their mitochondrial β-oxidation above their cytoplasmic esterification, with the corresponding negative consequences described for glucose-induced insulin secretion. It has been proposed by J. D. McGarry that in nonlipogenic tissues like heart and skeletal muscle, malonyl-CoA acts as a “fuel sensor” whose primary role is to regulate the rate of fatty acid oxidation [62]; this may also be applied to β-cells. Under regular fed conditions, plasma FFAs may suffer intermittent but not chronic elevations, except in obese and diabetic patients; in short-term expositions to elevated plasma FFA levels, predominates their capacity to potentiate glucose-induced stimulation of insulin secretion through an increase in their esterification and synthesis of lipidic signaling molecules, facilitating cellular Ca^2+^ uptake and its intracellular redistribution. In long-term exposition of β-cells to high FFAs (obese and type 2 diabetic patients), an excessive accumulation of FFA/TG in tissues may contribute to lipotoxicity and degradation of fat and muscle cells [62].

An interesting finding is that palmitate metabolism is under the negative regulation of norepinephrine through activation of α_2_-adrenergic receptors and the stimulation of a guanylyl cyclase via mediation of a G_02_ effector protein that increases the cytosolic cGMP concentration. This capacity of cGMP to suppress islet palmitate metabolism through inhibition of fatty acid synthetase (FAS) leads to blocking both FFA oxidation and esterification [31]. It is unknown whether norepinephrine also inhibits acyl-CoA carboxylase (ACC) through the same mechanism in islet β-cells or other tissues. Anyhow, BrS is a safe and effective inhibitor of ACC that suppresses FFA oxidation favoring esterification [4]. These two inhibitors might allow us to check in vitro whether increased FFA esterification causes β-cell lipotoxicity and the suppression of whole FFA metabolism (FAS inhibition) prevents it. We could check whether own FFAs, not their HS-CoA derivatives, might also participate in β-cell degradation via its chronic application. Nowadays, the FFA concentration at which the fatty acyl-CoA synthetase (FAS) enzyme becomes saturated has not been settled; islet [U-^14^C] palmitate oxidation to ^14^CO_2_ seems to be almost saturated at 1 mM [4]. There seems to be no consensus regarding the priority of lipo- over glucotoxicity or vice versa [62]. Considering the possibility of preventing or retarding the effects of lipotoxicity, the guanylyl cyclase of β-cells might be a good target for pharmacological therapy.

## 3. Implication of FFA Metabolism in the Glucagon Secretory Function of Pancreatic α-Cells

### 3.1. Introduction

It is understandable that knowledge of the stimulus–secretion coupling mechanism of glucagon secretion lags behind that of insulin due to the scarcity of the alpha cell population in the minimal physiological context of isolated pancreatic islets [63]. This restricts the use of biochemical and other technologies in the study of their function. In this review, we are focusing on the possible contribution of FFA metabolism to the modulation of glucose regulation of glucagon release. Several recent reviews are recommended that represent different interpretations of the stimulation of glucagon secretion by different nutrient secretagogues [64,65,66,67,68], which are not the focus of this review.

The most accepted view is that plasma glucose levels determine the degree of glucagon release in an inverse way to that observed for insulin: the higher the glucose concentration, the lower the glucagon secretion, and vice versa [69]. When plasma glucose concentration rises, basal (at 5 mM glucose) glucagon release is suppressed to lower levels. α-cells possess a glucose-recognizing mechanism like that of β-cells (K_ATP_^+^- and voltage-dependent Ca^2+^-channels), and it might be expected that glucose-induced inhibition of glucagon secretion would happen via reversal of its stimulatory mechanism. This would require an activation of the α-cell K^+^_ATP_ channel by ATP at a high glucose concentration, which is contradictory with the homologous mechanism of glucose-induced secretion of the β-cell (a “K^+^_ATP_ paradox”). It may be concluded that inhibition of glucagon secretion by high (>5 mM) glucose occurs via a different mechanism than the reversal of its stimulation in a lower range of glucose concentrations (from 0 to about 5 mM).

This is not the sole enigmatic question to be solved. There is widespread agreement that blood glucagon levels are increased in diabetic patients despite the large increase in their blood glucose values [69]. Is there an additional mechanism of glucagon secretion stimulation responsible for this increase in plasma glucagon concentration?

### 3.2. α-Cell Glucose Metabolism and Its Implication in the Regulation of Glucagon Secretion

In the periods of starvation between regular meals, glucagon secretion is stimulated by glucose in a restricted range of the sugar concentration from 0 to about 5–7 mM glucose, above which inhibition takes place [70,71,72,73]. The half-maximal concentration for the stimulation of glucagon secretion by glucose was evaluated to be around 3 mM [71]. The ATP content of α-cells [70,74] and αTC1-9 cells [73] increases with the glucose concentration, but only in a low range (1–10, 1–5, and 1–3 mM, respectively). At 5 mM glucose, the ATP/ADP ratio (measured with PercevalHR) was significantly lower in β- than α-cells; lowering glucose to 1 mM decreased the ratio in 69% of β-cells, but only in 94% of α-cells [74]. Similarly, the ATP/ADP ratio was increased by a rise of glucose from 1 to 6 mM glucose; it declined to previous values after the glucose concentration was again reversed to 1 mM [75]. Representative parameters of metabolism measured as ^14^CO_2_production in α-cells [70] and FAD content in islets [76] show poor or no glucose-dependent progression in α-cells. The resting membrane potential and the depolarization with 15 mM KCl of α-cells isolated from mouse islets pre-treated with alloxan were considerably greater and smaller, respectively, than in β-cells [77]. The depolarization effect of sulfonylureas on α-cells was very variable, although in inside-out patches of their plasma membrane, K_ATP_^+^ channels were blocked similarly to those of β-cells. The authors concluded that K_ATP_^+^ channel closure is a less dominant factor in the coupling of metabolic changes with electrical events in α-cells than in β-cells [77]. It would be interesting to know the effect of a range of glucose concentrations on the activity of K^+^_ATP_ channels in α-cells.

Although glucose uptake by α-cells is controlled by low Km transporters (GLUTI, SGLT1, and SGLT2) [78,79], it has been reported that they do not limit glucokinase activity; the measured rate of glucose utilization is about 10-fold lower than glucose uptake and is of similar magnitude in α- and β-cells [80]. However, whereas GLUT 2 in β-cells is a low-affinity and high-capacity glucose transporter, α-cells glucose transporters have high affinity and low capacity, which would favor glucose metabolism at a lower range of glucose concentrations than GLUT2. The experimental data published more recently (see paragraph above) do not confirm the view that α-cell glucose metabolism is performed in the same range of sugar concentrations as in β-cells. Therefore, a detailed kinetic study of both glucose utilization and oxidation by α-cells is needed to more precisely define two biochemical aspects: 1. the glucose concentration at which its utilization rate is saturated and the utilization/oxidation ratio. The maximum rate of glucose utilization will condition the maximum rate of lipogenesis, limited by the amount of pyruvate oxidized in the mitochondria. 2. Is α-cell glucose metabolism controlled by the glucose–fatty acid or the Randle cycle [2]? This regulatory cycle explains the regulation of glycolysis and glucose oxidation by fatty acid oxidation, mainly mediated by product (glucose-6-phosphate) inhibition of low Km hexokinase after previous inhibition of phosphofructokinase by an increased citrate concentration generated in the citric acid cycle, consequent to increased FFA oxidation; an accompanying elevation of the acetyl-CoA/Coa ratio activates pyruvate dehydrogenase kinase that phosphorylates pyruvate dehydrogenase in its inactive form [1]. Its confirmation would require an experimental demonstration of FFA inhibition of α-cell glucose utilization. Like in β-cells, glucokinase is the rate-limiting enzyme of glycolysis in α-cells [80], and it is not feedback-inhibited by its product; therefore, one might expect that, like in β-cells, α-cells might not be controlled by the glucose–fatty acid cycle [4].

### 3.3. Effect of Starvation on Glucose Regulation of Glucagon Secretion

After starvation of rats for 48 h, their isolated and perifused islets showed an almost complete suppression of their insulin response to 20 mM glucose, whereas the basal (3 mM glucose) rate of glucagon secretion was not decreased by high glucose [81]: this suggests that glucagon inhibition might be an indirect or direct effect of insulin, but not of glucose. Inhibition of palmitate oxidation by 2-bromostearate (0.25 mM BrS) completely restored glucose-induced insulin secretion in “starved” islets without modifying its basal release (at 3mM), but it increased several-fold the basal glucagon release, which was anyhow inhibited after elevating the glucose concentration to 20 mM [81]. In “fed” islets, BrS did not modify either basal or 20 mM glucose-induced insulin secretion, but it blocked glucose-induced inhibition of glucagon release, increasing it to the previous level registered at 3 mM glucose. It may be inferred that, in this case, BrS effect on glucagon secretion was not dependent on either glucose or secreted insulin. These two latter assertions suggest the possibility that glucagon secretion may be stimulated by inhibition of FFA oxidation, besides elevations of either glucose or insulin release. Basal somatostatin secretion was also increased around 2-fold, with a small initial peak.

Another piece of experimental evidence supporting the participation of increased FFA anabolism in glucagon secretion is provided by the direct effect of palmitate on islet hormone secretion. Palmitate (0.5 mM), in the presence of 6 mM glucose, triggered a significant and transitory insulin secretory response, but it had no stimulatory effect on the secretion of α- and δ-cells measured simultaneously. The combination of 0.5 mM palmitate plus 0.25 mM BrS transformed the insulin transitory response to palmitate alone into a sustained release that fell abruptly after decreasing the glucose concentration to 3 mM [81]. In the same experiments, glucagon release was strongly stimulated and did not return to basal values after decreasing the glucose concentration. Somatostatin secretion followed a similar pattern to that of insulin, but of minor intensity.

### 3.4. Precedents of the Effects of FFA Metabolism on the Regulation of Glucagon Secretion

The described potentiating effects of palmitate on the three islet hormones’ secretion confirm previous work that also demonstrated its dependence on FFA uptake by the corresponding islets cells through G-coupled fatty acid receptors (FFAR/GPR40) [82]. Using specific FFAR1 antagonists, the authors concluded that transported FFAs had to be metabolized to stimulate hormone secretion, but they did not provide evidence of the metabolic pathway being coupled to the stimulation of hormone secretion. An older report confirmed that palmitate (0.5 or 1.0 mM) enhanced glucagon secretion in mice islets at 1–15 mM glucose, attributing it to an elevation of intracellular free calcium [83].

A recent report showed that inhibition (TOFA, 5-(tetradecyloxy)-2-furoic acid) or specific knockdown of ACC1 in α-cells of mouse islets (gluACC1KO islets) impaired the rate of glucagon secretion at 1 mM glucose as compared with control islets [84]. Fasting serum glucagon levels were elevated by fasting and decreased within 50% in control animals in an IPGTT, whereas gluACC1KO mice exhibited lower fasting glucagon levels that were not further decreased by glucose. In control α-cells, K_ATP_ channel conductance was significantly elevated at 1 mM glucose relative to 6 mM, and it was reduced in gluACC1KO α-cells. The later cells also showed diminished (−50%) depolarization-driven Ca^2+^currents. The authors found that S-acylation of the K_ATP_ channel subunit Kir6.2 took place in α-cells cultured for 22 h with 15 mM glucose in the absence and presence of TOFA. The ACC1 inhibitor induced a consistent but non-significant increase in the S-acylation of the Kir6.2 subunit. 2-bromopalmitate (2-BP, 0.1 mM) was intended to be used as a blocking agent of S-protein acylation to check its effects on glucagon secretion. 2-BP (also a known inhibitor of CPTI enzyme, homologous to BrS [5]) induced a strong stimulation of glucagon release in primary islets with 1 mM glucose. The authors concluded that “2-BP treatment demonstrates that protein Kir6.2 acylation pays a role in glucagon secretion”. However, they obviated to comment on the possible contribution of 2-BP (2-bromostearate analogue)-dependent inhibition of FFA oxidation and increase in FFA esterification to glucagon and insulin secretion, as has been published before [3,4,81]. However, the work offers strong supporting evidence for the dependence of glucagon release on FFA esterification, as demonstrated by the strong stimulation of glucagon secretion by 2-BP.

Recent experimental evidence suggests that inhibition of mitochondrial FFA oxidation by glucose is responsible for the suppression of glucagon release via a decrease in cellular ATP and membrane repolarization in α-cells [85]. It is difficult to understand why murine α-cells in islets preincubated for 1 h with 1 mM glucose were disabled, decreasing their glucagon release during a further incubation with 5 mM glucose, unless 0.36 (or higher?) mM FFAs were present. By contrast, preincubation with 5 mM glucose did not require addition of FFAs to suppress glucagon secretion in the subsequent incubation with 5 mM glucose; neither were FFAs required to suppress glucagon secretion after changing the glucose concentration from 3 to 20 mM. Moreover, the authors have not demonstrated that an increase in glucose concentration lowers FFA oxidation in α-cells (Figures 1E and 4L). β-oxidation was measured in intact wild-type and transgenic (αCPT 1aKO) islets, but not in α-cells. As shown in Figure 4L, knock out of the αCPT1a gene did not significantly diminish β-oxidation in transgenic islets as compared to wild-type islets. The observed reduction in β-oxidation in both types of islets is therefore attributable to the inhibition of CPT 1a activity by G5 in β- but not in α-cells.

The observed suppression of ATP in α-cells of murine islets by 5mM mM glucose contradicts previous studies, as mentioned two paragraphs above [70,73,74,75], supporting an increase in glucose metabolism, intracellular ATP, and glucagon secretion in the low range of glucose concentrations (0 to 5 mM). Might it be due to a long-term pre-exposition to an unphysiological glucose concentration (1 mM)? One might conclude that the presence of FFA is not strictly required for glucose-induced inhibition of glucagon secretion.

It would be interesting to further characterize the interaction of glucose and FFA in α-cells in more detail. One concern is authors’ interpretation of the function of the glucose–fatty acid cycle. As mentioned above, it was designed to explain the inhibition of glycolysis by FFAs during fasting/starvation, not the inhibition of FFA oxidation by glucose [2]. Therefore, one should demonstrate that FFAs inhibit islet glycolysis to conclude that the cycle is operative in alpha cells. Specific overexpression of pyruvate dehydrogenase kinase 4 (PDK4) was shown to suppress glucose reduction in cellular ATP and glucagon release [85], as expected. Blocking pyruvate dehydrogenase activity by its hyperphosphorylation turns it inactive, impairing the citric acid cycle via a shortage of acetyl-CoA derived from glucose and not FFAs, resulting in diminished ATP production. Therefore, PDH suppression cannot be considered a specific inhibition of lipogenesis because it interferes with multiple mitochondrial processes.

Etomoxir (100 µM), an irreversible CPT 1a inhibitor, causes inhibition (−40%) of glucagon secretion with 1 mM glucose, as well as the ATP/ADP ratio in mouse α-cells and palmitate oxidation in αTC1-6 cells [86]. General knockout of CPT1a in mice did not affect plasma glucagon concentration: it induced a modest decrease in fasting plasma glucose and had no effect on the protein expression of two gluconeogenic enzymes. Etomoxir (100 µM) reduced action potential amplitude and partially depolarized the α-cell plasma membrane, and similar effects were reproduced in αCPT1a-KO cells without affecting G_KATP_. The resulting paradox—that the membrane could be depolarized after a relevant decrease in α-cell ATP—was explained by a reduction in the plasma membrane electrogenic Na^+^-K^+^ pump due to reduced production of mitochondrial ATP, promoted by a suppression of FFA oxidation. In fact, 0.5 mM ouabain decreased the action potential amplitude with 1 mM glucose and glucagon secretion, but it did not modify the conductance of the K_ATP_^+^ channels. One concern is the relatively high etomoxir dose used, given that it has off-target effects above a 5 µM concentration: it can affect oxidative metabolism of substrates that are different from FFAs, and it interferes with respiratory complex I. Lipidomic profiling of isolated mitochondria from CPT1A cells shows relevant decreases in the amounts of many complex lipids species, besides other mitochondrial morphological and biochemical changes [53,54].

### 3.5. Experimental Evidence in Favor of the Predominance of FFA Anabolism over Catabolism on the Stimulation of Islet Insulin and Glucagon Secretion

1. 0.25 mM 2-bromostearate (BrS) has no effect on basal and glucose-stimulated insulin secretion of “fed” islets [81].
2. 0.25 mM 2-bromostearate (BrS) blocks glucose-induced inhibition of glucagon release in “fed” islets [81].
3. 0.25 mM BrS recovers the complete suppression of glucose-induced insulin secretion of “starved” islets [81].
4. 0.1 mM 2-bromopalmitate (BP, homologous of BrS) stimulates glucagon secretion with 1 mM glucose around 5-fold in mouse islets [84].
5. Deletion of the acetyl-CoA carboxylase gene codifying the enzyme responsible for malonyl-CoA synthesis significantly lowers fasting plasma glucagon levels [84].
6. 0.25 mM BrS, in the presence of 6 mM glucose, converts a transient stimulation into a sustained stimulation of palmitate-induced insulin secretion [81].
7. Under the same conditions as above, basal glucagon secretion was stimulated within approximately 50%, and it did not return to basal values after withdrawing the stimulus [81].

### 3.6. Possible Metabolic Mechanisms Associated with the Regulation of Glucagon Secretion

1. FFA oxidation by β- and α-cells does not contribute to the stimulation of insulin and glucagon secretion in the presence of glucose. FFA anabolism to complex lipids, rather than its catabolism, is responsible for the potentiation of glucose activation of hormone secretion.
2. β-cell secretion is more sensitive to the stimulation of FFA anabolism than α-cell secretion. This probably depends on a higher flux of β-cell lipogenesis than α-cell lipogenesis within the range of glucose concentrations that stimulate insulin release and inhibit FFA oxidation (from 6 to 20 mM glucose). This is concordant with the lack of BrS effect on glucose-stimulated insulin secretion in “fed” islets and its capacity to restore insulin secretion in “starved” islets to the level of their “fed” controls with further stimulation. In other words, BrS partially plays the role exerted by high glucose. In support of this view, palmitate stimulates a transient stimulation of insulin secretion at a low glucose concentration (6 mM) that is changed into a sustained release by BrS, probably because at this glucose level, the synthesis of malonyl-CoA is not as high as at greater sugar concentrations.

α-cells, which are stimulated in a lower range of glucose concentrations than β-cells, might need a higher availability of plasma FFAs to increase their anabolism because of their lower rate of lipogenesis. Accordingly, in “starved” α-cells, BrS stimulates basal glucagon secretion several-fold above the control basal level of “fed” islets. This excessive glucagon secretion in response to BrS suggests that, under physiological conditions, α-cells are never exposed to an inhibition of FFA oxidation as extreme as that of β-cells, although they might be stimulated by higher-than-“basal” FFA concentrations. This might explain the hyperglucagonemia observed in glucose-intolerant and diabetic subjects. It could be due to a stimulation of their glucagon secretion by a several-fold-higher plasma FFA concentration than what exists in healthy people, as well as the possible contribution of CPT1a that is less sensitive to being inhibited by malonyl-CoA in the context of hyperglycemia and hypo-insulinemia, like what happens in the liver [7].

3. Blocking of the inhibitory effect of high glucose on glucagon secretion by starvation is synonymous with a stimulation of glucagon secretion at (not “by”) a high sugar concentration. Suppression of β-cell insulin secretion by glucose during starvation probably avoids the threat of hypoglycemia from elevated FFA plasma levels. More mechanistically, it suggests that the failure of an intra-islet hormonal action of insulin on glucagon secretion might be responsible for the restoration of glucagon release to fed control rates. We have no expert opinion on the open discussion about the mechanism responsible for the interaction between β- and α-cells, which is mediated by either a vascular mediation of secreted insulin from the β-cell core to the α-cells in the mantle (“core–mantle view”) to inhibit them or by a mantle-to-core diffusion of secreted glucagon to stimulate insulin secretion (paracrine view) [87,88]. However, we are prone to the “core–mantle” hypothesis, given that in isolated islets of starved animals, the “only” iatrogenic intervention was islet isolation, and the cause of islet functional changes was a physiological regulation of FFA body metabolism that resulted in the failure of glucose to decrease basal glucagon secretion in the absence of an insulin response to glucose. In a more naïve view, if the respective rates of secretion of insulin (ng/islet/min) and glucagon (pg/islet/min) secretion are expressed in molar units/islet/minute in one of our islet perifusion experiments, the insulin rate exceeds that of glucagon by around 6-fold, and the core-to-mantle gradient of insulin is higher than that of glucagon in the opposite direction, from the mantle to the β-cell core.

**Figure 3:**
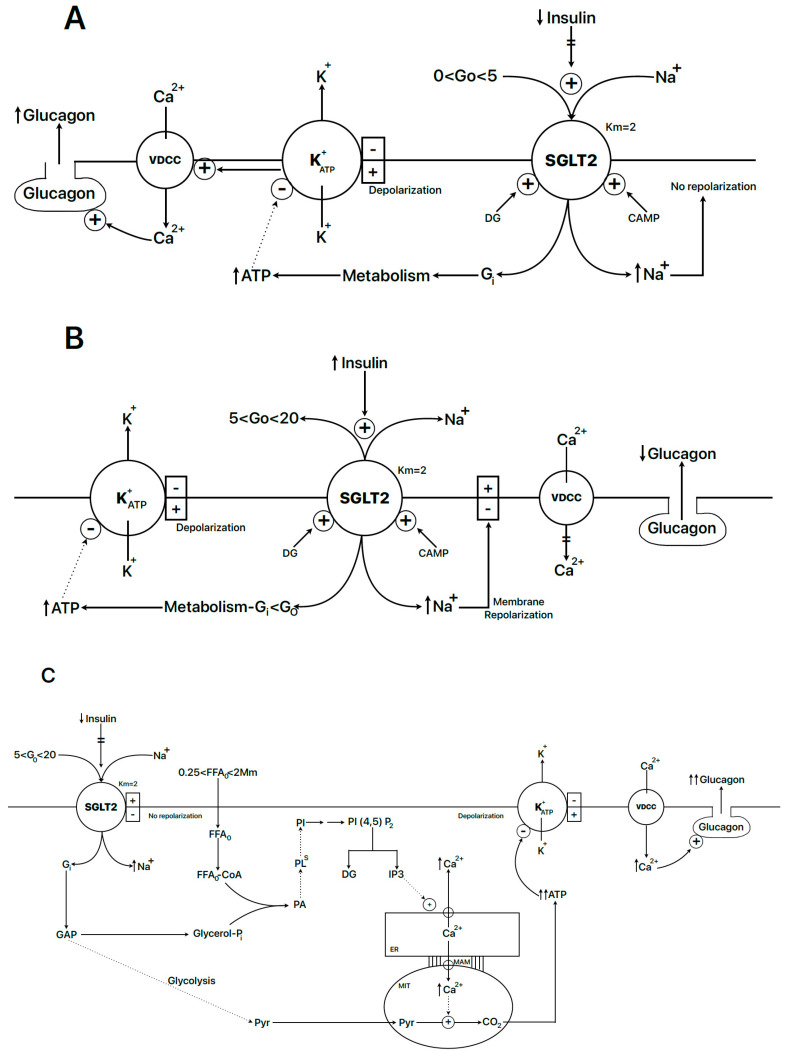
(**A**). Hypothetical stimulus–secretion coupling mechanism of glucagon secretion by glucose in the range from 0 to 5 mM. Sodium–glucose cotransporter 2 (SGLT2), voltage-dependent calcium channel (VDDC), diacylglycerol (DG). (**B**). Hypothetical mechanism of inhibition of glucagon secretion via stimulation of insulin secretion in the glucose range from 5 to 20 mM. Symbols are the same as in (**A**). (**C**). Hypothetical stimulus–secretion coupling mechanism of glucagon secretion by glucose in the range from 5 to 20 mM under hyperglycemic, hypo-insulinemic, and hyperlipidemic conditions (glucose intolerance and diabetes). It is assumed that plasma FFAs are close to 2 mM. Inositol (3,5), trisphosphate (IP3). Other symbols are the same as in (**A**,**B**).

In summary, the regulation of changes in α-cell glucagon secretion probably occurs by means of three mechanisms: 1. glucose stimulates glucagon secretion in a low range (1 to 5 mM?) (Figure 3A). 2. Glucose-induced insulin secretion in the range 5 to 20 mM, indirectly or directly inhibits glucagon secretion (Figure 3B). 3. Glucagon is stimulated by an elevation of FFA concentration and its anabolism in α-cells in the context of hyperglycemia, hyperlipidemia, and hypo-insulinemia, like glucose intolerance or diabetes (Figure 3C).

### 3.7. Mechanism of Inhibition of Glucagon Secretion by Co-Secreted Insulin (Core-to-Mantle View)

There are contradictory reports about a hypothetical direct mechanism of glucagon release inhibition via a stimulatory glucose concentration. However, it has been demonstrated that glucagon-secreting cells, like In-R1-G9 and αTC clone 6 cells, possess functional insulin receptors [89]. Islets from cell-specific insulin receptor knockout (αIRKO) mice [90] or downregulation of insulin receptor (IR) expression with siRNA-IR [91] in In-R1-G9 cells exhibit a significant suppression of glucose-induced inhibition of glucagon release. αIRKO mice show glucose intolerance, hyperglycemia, and hyperglucagonemia in the fed state [90].

Co-cultured insulin secreting (MIN6) with glucagon-releasing (αTC6-1) cells led to two conclusions [92]: 1. αTC6-1 glucagon release was suppressed in co-culture with MIN6 cells, the latter being scattered in the flask. 2. Glucagon secreted by αTC6-1 cells did not affect insulin release by MIN6 cells. Similar results were obtained with human islet cell populations of α- and β-cells [93]; glibenclamide decreased the release of glucagon only in both types of co-cultured cells, but not in a single α-cell culture. Somatostatin release was stimulated at 16.8 mM glucose in whole islets, but not in mixed aggregates of islet cells [93].

### 3.8. Hypothetical Mechanisms of Inhibition of Glucagon Secretion via Insulin-Mediated Stimulation of Somatostatin Secretion or a Direct Inhibitory Action of Secreted Insulin

A pioneering work showed that glucose-stimulated pulsatile secretion of insulin and somatostatin in the perfused rat pancreas were almost synchronous [94]: somatostatin pulses lagged slightly (30 s) behind those of insulin. Glucagon oscillations were asynchronous with insulin; they had a delay of 2.4 ± 0.1 min with respect to somatostatin oscillations and peaked progressively lower with the rise of somatostatin. An antagonist of purinergic receptors P2Y_1_ (1 µM MR 2179) abolished somatostatin and glucagon pulses, but insulin pulses were maintained. At 1 µM MR 217, its effects were reversible, but not at a 10-fold higher concentration. In perifused mice islets stimulated with either tolbutamide (500 µM) or KCL (15 mM), the rise in insulin release preceded that of glucagon [94].

The recently enunciated hypothesis on the mechanism responsible for insulin suppression of glucagon release (glucagonostatic effect) at high concentrations of glucose implicates a simultaneous increase in δ-cell secretion [95]: insulin stimulates somatostatin secretion considerably with 4 mM glucose (>200%) that was abolished by an insulin receptor antagonist (S961); surprisingly, somatostatin stimulation was not saturated at insulin concentrations as high as 0.3 µM. Somatostatin suppression of glucagon release was blocked by a somatostatin receptor antagonist (CYN154806). In islets isolated from transgenic mice with a specific knockout of the δ-cell insulin receptor (SIRKO mice), insulin (100 nM) failed to stimulate somatostatin secretion and to suppress glucagon release. Insulin was earlier shown to act as an agonist of the glucose–sodium cotransporter SGLT2, increasing its maximum rate but not its glucose affinity [96]. Therefore, studies tested whether this transporter had any functional role on insulin-dependent somatostatin secretion. Either decreasing the extracellular Na^+^ concentration to 10 mM or adding a SGLT2 inhibitor (dapaglifozin 0.1 or 12.5 nM) practically abolished the inhibition of glucagon secretion by insulin [95].

Human Na^+^-glucose cotransporter (hSGLT2), expressed in human embryonic kidney (HEK) 293T cells, has a sodium/glucose coupling ratio of 1:1; together with hSGLT1, it is an electrogenic transport protein energized by the plasma membrane electrochemical potential, generating a Na^+^-glucose inward current in α-cells [96]. hSGLT2-mediated maximum transport of D-[α-methyl-^14^C]glucopyranoside ([^14^C]α-MDG) was increased 225% and 150% by 0.1 mM 8-Br-cAMP (PKA activator) or 1.0 µM *sn*-1,2-dioctanoylglycerol (PKC activator), respectively. Insulin induced a 72% increase in [^14^C]α-MDG at 100 pM and a 2.3-fold increase at 400 pM, abolished in S624A-mutated hSGLT2 [96]. This experimental evidence supports the alternative possibility that insulin might directly mediate the suppression of glucagon secretion by polarizing the α-cell membrane, due to an increased influx of extracellular sodium together with glucose that might counteract the effect of K^+^_ATP_ channel closure at a >5 mM glucose concentration and avoid voltage-dependent calcium channel activation. It would be interesting to know whether insulin acting through its classical mechanism, the PI3K-AKT pathway, is also responsible for the translocation of GLUT4 to the plasma membrane in other cells.

### 3.9. Potentiation of Insulin Secretion by Glucagon or Incretin Peptides (Paracrine or Mantle-to-Core View)

In the paracrine (or mantle-to-core) view, it is assumed that glucagon or the two incretin peptides, glucagon-like peptide (GLP1) and glucose-dependent insulinotropic polypeptide (GIP), are released from islet α-cells to activate specific G-protein coupled receptors in β-cells, contributing to potentiating glucose-induced insulin secretion. GLP1 is a product of preproglucagon processing that is released from the α-cell beside the intestinal L cells [66]. The GIP receptor (GIPR) is expressed in both α- and β-cells [97]. Knockout of β-cell receptors to GIP in α-cells suppressed the potentiation of alanine stimulation of glucagon secretion by 10 nM GIP. The final interpretation is that GIP potentiation of alanine-induced glucagon secretion improves insulin secretion in turn [97]. Transcription inhibition of intestinal, pancreatic, and brain preproglucagon strongly suppressed the glucose response to a mixed meal [98]. Reactivation of preproglucagon transcription in the pancreatic territory but not in the intestine provoked higher glucose excursions in an OGTT that were reduced in the presence of exendin (9–39), pointing to the intervention of GLP1 [98]. Glucagon (0.1, 1.0, and 10 nM) stimulates insulin secretion in perfused mouse pancreata with 12 mM glucose [99]. Knockdown of both β-cell-specific glucagon and GLP1 receptors was necessary to induce an almost complete suppression of insulin secretion [99]. With the premise that a mixed meal stimulates both insulin and glucagon, a later report supported the previous experimental results: double β-cell-specific knockout of glucagon and GLP1 receptors blocked glucagon (1 mg/Kg) capacity to reduce glycemia and to stimulate insulin secretion, compared with wild-type animals [100]. A “designer receptor exclusively activated by designer drugs” (DREADD), derived from a mutated M_4_ muscarinic receptor (hM4Di) activated by clozapine N-oxide (CNO), was inserted in the preproglucagon gene under the control of a strong CAG promoter separated from the hM4Di coding sequence by a loxP site-flanked Stop signal [101]. Crossing this transgenic mouse with another carrying a Cre-Gcg allele controlled by tamoxifen generated an “α-GID” mouse strain. Addition of tamoxifen activated the Cre recombinase, cutting the Stop signal that allowed preproglucagon gene transcription. Subsequent activation of the hM4Di receptor with CNO in α-GID mice almost completely suppressed plasma glucagon concentration in a time interval and significantly reduced plasma insulin concentration without affecting plasma glucose values [101]. An IGTT (2 g/Kg i.p.) in α-GID mice treated with CNO (1 mg/Kg i.p.) showed impaired glucose tolerance—greater excursions of blood glucose and strong reductions in both glucagon and insulin. Glucagon addition (10 nM) rescued the suppression of insulin release triggered by an increase in the glucose concentration from 3 to 12 mM in perifused islets of α-GID mice. It can be concluded that a potentiation of insulin release by islet-secreted glucagon or/and incretin peptides may contribute to the potentiation of insulin release at elevated glucose levels, but not at low concentrations. It has been demonstrated that a mixed-nutrient meal induces an elevation of plasma glucagon levels in non-diabetic as well as T2D individuals, suggesting its possible role, together with incretin peptides, in glucose recognition in postprandial conditions [100].
